# Rates of thoracic trauma and mortality due to accidents in Brazil

**DOI:** 10.4103/1817-1737.44782

**Published:** 2009

**Authors:** Francisco Cury, André Luciano Baitello, Rodrigo Florêncio Echeverria, Paulo César Espada, José Maria Pereira de Godoy

**Affiliations:** *Department of Surgery, São José do Rio Preto University School of Medicine, São Paulo, Brazil*; 1 *Department of Trauma, São José do Rio Preto University School of Medicine, São Paulo, Brazil*; 2 *Professor of the Medical School in São José do Rio Preto (FAMERP) and Researcher for the National Council of Technological and Scientific Development (CNPq), Brazil*

**Keywords:** Mortality, prevalence, thorax, trauma

## Abstract

**AIM:**

To report on the causes of trauma, indexes of trauma, and mortality related to thoracic trauma in one region of Brazil.

**MATERIALS AND METHODS:**

This prospective study was performed at the Regional Trauma Center in São José do Rio Preto over a 1-year period, from 1^st^ July 2004 to 30^th^ June 2005. We included all patients attending the center's emergency room with thoracic trauma and an anatomic injury scale (AIS) ≥ 2. We collected data using a protocol completed on arrival in hospital utilizing the AIS. We studied the types of accidents as well as the mortality and the AIS scores. Prevalence rates were calculated and the paired t-test and logistic regression were employed for the statistical analysis.

**RESULTS:**

There were a total of 373 casualties with AIS ≥ 2 and there were 45 (12%) deaths. The causes of thoracic trauma among the 373 casualties were as follows: 91 (24.4%) car crashes, 75 (20.1%) falls, 46 (12.3%) motorbike accidents, 40 (10.7%) stabbings, 22 (5.9%) accidents involving pedestrians, 21 (5.6%) bicycle accidents, 17 (4.6%) shootings, and 54 (14.5%) other types of accident. The severity of the injuries was classified according to the AIS: 224 (60%) were grade 2, 101 (27%) were grade 3, 27 (7.2%) were grade 4, 18 (4.9%) were grade 5, and 3 were (0.8%) grade 6. With respect to thoracic trauma, pedestrians involved in accidents and victims of shootings had mortality rates that were significantly higher than that of those involved in other types of accidents.

**CONCLUSION:**

Road accidents are the main cause of thoracic injury, with accidents involving pedestrians and shootings being associated with a greater death rate.

Thoracic trauma is one of the leading causes of death in all age-groups and accounts for 25–50% of all traumatic injuries.[1] While the majority of patients with thoracic trauma can be managed conservatively, a small but significant number require emergency thoracotomy as part of their initial resuscitation.[1,2] Nevertheless, severe chest injuries are still associated with significant morbidity and mortality.[3,4]

Early surgery for chest injuries is often justified because it averts immediate threats to life (level 1c evidence); however, in selected hemodynamically stable patients delayed treatment is also possible.[3] Lesions of the tracheobronchial system and diaphragmatic ruptures should be treated urgently with primary surgical repair. Penetrating chest injuries in hemodynamically unstable patients require emergency operative therapy.

Disproportionate or unilateral lung involvement calls for more than just general supportive care. Independent lung ventilation (mostly used in cases of unilateral lung involvement) and other strategies like nitric oxide inhalation, prone positioning, partial liquid ventilation, and extracorporeal membrane oxygenation have given good results.[5,6] The need for immediate cardiopulmonary resuscitation after accidents is an predictor of high mortality and further studies need to be done to review the indications and the ethical aspects involved.

The causes of thoracic trauma may vary in different regions as they are influenced by the lifestyles of the population. The aim of this work is to report on the causes of trauma, indexes of trauma, and mortality related to thoracic trauma in one region of Brazil.

## Materials and Methods

This prospective study was performed in the Regional Trauma Center in São José do Rio Preto-SP-Brazil over a 1-year period, from 1^st^ July 2004 to 30^th^ June 2005, and included all patients reporting to the center's emergency room with thoracic trauma and an anatomic injury scale (AIS) score ≥ 2. Data were collected using a protocol completed on arrival in hospital utilizing the AIS. These data were analyzed using the Microsoft^®^ Excel, version XP/2003.

We examined the types of accidents as well as the mortality and the AIS.

The paired t-test and logistic regression were employed in the statistical analysis, with an alpha error of 5% being considered acceptable (*P*-value < 0.05).

## Results

The patients' ages ranged between 13 and 89 years, with a mean of 37.9 years and a standard deviation of 17.3. Seventy-four of the patients were women and 291 were men; this difference in numbers was statistically significant (*P* < 0.0001). The mean age of the women was 43.2 years (±20.0 years) and of the men it was 36.4 years (±16.3 years). The t-test demonstrated that women and older men had a statistically significant higher risk of being involved in accidents (*P* = 0.08).

There were a total of 373 casualties with an AIS ≥ 2, and there were 45 (12%) deaths. The causes of thoracic trauma among the 373 casualties were as follows: 91 (24.4%) car crashes, 75 (20.1%) falls, 46 (12.3%) motorbike accidents, 40 (10.7%) stabbings, 22 (5.9%) accidents involving pedestrians, 21 (5.6%) bicycle accidents, 17 (4.6%) shootings, and 54 (14.5%) other types of accident.

The severity of the injuries was classified according to the AIS, with 224 (60%) grade 2, 101 (27%) grade 3, 27 (7.2%) grade 4, 18 (4.9%) grade 5, and 3 (0.8%) grade 6 injuries.

With respect to the accidents causing thoracic trauma, pedestrians involved in accidents had a mortality rate significantly higher than others except for those injured in shootings (*P* < 0.004; relative risk = 2.9 ; 95% CI: 1.6–5.5).

With shootings, the mortality was significantly higher than in the other types of accidents (*P* < 0.0002; relative risk = 4.1; 95% CI: 2.4–7.0); however, as mentioned earlier, the mortality rate of victims of shootings was not significantly different from that of pedestrians involved in accidents. There were also no significant differences among the mortality rates in the other types of accidents. Table 1 shows the regression logistics of the index AIS and mortality of the head/neck, face, thorax, abdomen, extremities, and pelvis.

**Table 1:** Logistic regression of the index anatomic injury scale and the mortality

Predictor	Coefficient*	*P*-value	Odds ratio	95% CI
AIS (head/neck)	0.68	< 0.0005	1.97	1.52–2.54
AIS (face)	0.66	0.056	1.93	0.98–3.81
AIS (thorax)	1.78	< 0.0005	5.96	3.66–9.71
AIS (abdomen)	0.46	0.003	1.58	1.17–2.13
AIS (extremity/pelvis)	0.13	0.54	1.14	0.75–1.74
AIS (extremities)	0.70	0.23	0.49	0.161–1.57

* Constant - −8.88, AIS - Anatomic injury scale

## Discussion

This study presents epidemiological data related to thoracic trauma in a reference center in Brazil where accidents involving cars and falls are the two most common causes of trauma. The prevalences of the types of accidents may vary by region according to the lifestyle of the population. Larger cities, where traffic accidents and physical violence are more common, may experience more serious injuries. In this study, about half of the traumas were caused by road accidents. It is important to note that pedestrians involved in accidents and victims of shootings were more likely to die than those involved in other kinds of accidents. Also, males and younger individuals were more likely to suffer thoracic trauma.

Mortality in these victims is associated to the trauma index where it is obvious the type of trauma influences the severity. Logistic regression shows that mortality could be explained by the severity of the lesion in the thorax and in lesions of the head and neck, face, and abdomen. Knowledge of the prevalence of different types of trauma in different regions may be of help for designing regional accident prevention policies. The organization of regional trauma centers can help to identify regional data in order to adapt to the local reality. In this study, the injuries caused by bicycle accidents were common and suggests that daily activities such as this may not be receiving adequate attention in injury prevention programs.

## Conclusion

Road accidents are the main cause of thoracic injury, with accidents involving pedestrians and shootings being associated with a greater death rate.
